# Role of nutritional supplements on oral health in adults – A systematic review

**DOI:** 10.12688/f1000research.134299.1

**Published:** 2023-05-15

**Authors:** Aishwarya Shodhan Shetty, Ramya Shenoy, Parul Dasson Bajaj, Ashwini Rao, Aparna KS, Mithun Pai, Avinash BR, Praveen Jodalli

**Affiliations:** 1 Department of Public Health Dentistry Manipal College of Dental Sciences Mangalore, Manipal Academy of Higher Education, Manipal, Karnataka, 575001, India

**Keywords:** Adults, Health, Nutrition, Nutritional supplements, Oral health

## Abstract

**Purpose:** The word ‘diet’ usually encompasses the consumption of food that includes all the necessary nutrients required for the optimal functioning of the body. Nutritional supplements hold a supreme position due to the demanding lifestyles or medical conditions in this current era. Although medical literature has sufficient evidence exploring the effect of nutrients on general health, this systematic review aimed at specifically evaluating the role of nutritional supplements on oral health in adults.

**Methods:** PRISMA guidelines were followed in conducting this systematic review where four electronic databases including Scopus, PubMed, EMBASE, and Web of Science were reviewed. Based on the inclusion criteria, four studies exploring the effect of oral nutritional supplements on oral health among the adult population were included in this systematic review.

**Results:** This review generated evidence suggesting a positive effect of nutritional supplements on oral health. These findings were depicted in the form of a reduction in plaque scores, probing depths, gingival inflammation, and greater improvement in periodontal healing among individuals with higher intakes of nutritional supplements within recommended doses.

**Conclusions:** The systematic review outlines the positive impact of consuming nutritional supplements in the recommended dosage on oral health. Additionally, this review stresses the necessity for interventional studies to further investigate the effects of nutritional supplements on oral health, particularly in regard to periodontal healing.

**PROSPERO registration: **CRD42021287797 (27/11/2021).

## Introduction

The term “diet” generally refers to the entirety of the food that a person consumes, with the implication that it includes all the necessary nutrients essential for the body ( Leech *et al.*, 2015). Nutrition plays a vital role in meeting the fundamental requirements for maintaining good health, impacting both overall wellness and oral health significantly. Essential nutrients are chemical substances in food which cannot be synthesized in our body at all or in sufficient amounts but are vital for life, growth and tissue repair ( Leaf & Weber, 1987; Mann & Truswell, 2017). It has been noted that taking essential nutrients before undergoing surgery and maintaining them at optimal levels can significantly enhance the effectiveness of the treatment. ( Miresmaeili *et al.*, 2015). This makes diet and nutrition an indispensable component in patient care ( Sobotka & Forbes, 2019).

Various dietary nutrients play a significant role in the maintenance of oral health. Vitamin A plays a role in preserving the mucosal membranes, salivary glands, teeth, and protecting against cleft palate ( Purvis *et al.*, 1973). Despite the prevalence of vitamin A deficiency in developing countries ( Haslam, 2019), it's important to recognize its positive effects on oral health. Vitamin E, with its anti-inflammatory action, helps to decrease the effect of oxygen-free radicals in the cells during the process of bone formation, thus having a positive impact on tooth formation ( Esenlik *et al.*, 2012; Sufarnap *et al.*, 2020). Vitamin D plays a crucial part in absorption of calcium, magnesium and phosphorus from gut and insufficiency of vitamin D might lead to enamel and dentin hypoplasia and delayed eruption ( Adegboye *et al.*, 2010; Lowenstam & Weiner, 1985). Vitamin K has an association with fetal facial deformity ( Howe & Webster, 1994). The role of ascorbic acid has been studied extensively in relation to collagen synthesis and therefore, vitamin C deficiency eventually leads to scurvy, which causes inflammation of the gingiva, commonly seen in populations who have limited intake of food like the elderly, and others include smokers and alcoholics ( Onigbinde OO *et al.*, 2021; Van Rooij *et al.*, 2003). The marginal deficiency of ascorbic acid can also cause periodontitis ( Alvares *et al.*, 1981; Krasse, 1960). It has been observed that a daily intake of a 1 gram supplement of vitamin C was significantly associated with a decrease in sulcular epithelial permeability and an increase in collagen formation in humans ( Leggott *et al*., 1986).

Deficiency of folic acid is usually said to have its association with neural tube defects and a reduction in cleft lip/cleft palate has been observed when pregnant women take folic acid supplement ( Burgess *et al.*, 2018; Van rooij *et al.*, 2004;). Folic acid deficiency also impairs the sulcular barrier function ( Burt, 1982; Dhamo *et al.*, 2021), and few studies have suggested that supplementation with folate might help in reducing the gingival fluid flow as well as the bleeding index. Vitamin B12 is known to contribute towards mucosal wound healing and facilitating bone health, both of which are necessary for recovery in cases of periodontitis. This fact is backed by studies which have demonstrated an inverse association between vitamin B12 and tooth count ( Hugar *et al.*, 2017; Zong *et al.*, 2016). Calcium and vitamin D have a significant association with bone mineralization and osteoporosis where mineralization defects are seen in severe deficiency of vitamin D, while calcium causes negative calcium balance and bone loss. This loss of bone is apparent not only throughout the body but also in the alveolar bone ( Adegboye *et al.*, 2010; Jimenez M *et al.*, 2014; Lowenstam & Weiner, 1985). Zinc is known to decrease the sulcular epithelial permeability by inhibiting the leukocyte activity, thereby decreasing gingival fluids and hence the gingival inflammation ( Garcia *et al.*, 2011; Rapisarda & Long, 1981; Vratsanos & Mandel, 1985). Diet is also known to have a considerable contribution towards prevention of particular oral tumors ( Morse, 2005). There is evidence which suggests that individuals who consume adequate amounts of vegetables, whole grain foods and fruits, have a 40–80% lowered risk of developing oral or pharyngeal cancers in comparison to people who have a lower consumption of these food types ( Franceschi *et al.*, 1999; Mclaughlin *et al.*, 1988).

There is a lack of literature on the consumption of nutrients and balanced diet on oral health. There are relatively fewer studies which explore the possible effects of oral nutritional supplements on oral health. Therefore, this systematic review aimed to evaluate the role of nutritional supplements on oral health in the adult population.

## Methods

This systematic review was conducted as per the Preferred Reporting Items for Systematic reviews and Meta-Analyses (PRISMA) ( Shenoy, 2023) statement and the literature search was started in April 2021 and concluded in October 2021. The protocol of the present systematic review was registered with PROSPERO ( CRD42021287797) (date of registration-27/11/2021).

### Study selection

A complete literature search was performed across four electronic databases i.e. Scopus, PubMed, EMBASE and Web of Science. Peer-reviewed literature, either published in English language or with availability of translated text in English, were included for the present systematic review. The papers published in or after 2010 and which met the pre-determined inclusion and exclusion criteria were included in this systematic review.

Inclusion Criteria: Randomized controlled trials and cross-sectional studies with outcomes based on the effect of nutritional supplements on plaque score, gingival health and periodontal health among adult population were included in the present systematic review. Papers with full text access and either published in English language or with availability of translated text in English were included in this review.

Exclusion criteria: Studies on children and animal studies were excluded along with studies which utilized an intravenous mode of administration for nutritional supplements. Studies which explored the effect of nutritional supplements on general health as well as unpublished papers or study protocols were excluded from this review.

### Search strategy

The search strategy followed across the four databases is presented in Table 1. Initially, the PubMed database yielded 235 search results, five articles were later excluded when a BOOLEAN term “NOT COVID” was added.

**Table 1.** Search Strategy across the databases.

Database	Search Terms
Scopus	(nutritional AND supplements AND oral AND health AND adults)
PubMed	((((Nutritional) AND (supplements)) AND (oral)) AND (health)) AND (adults) NOT COVID Filters: Free full text, Clinical Trial, Meta-Analysis, Randomized Controlled Trial, Review, Systematic Review
EMBASE	nutritional:ti,ab,kw AND supplements:ti,ab,kw AND oral:ti,ab,kw AND health:ti,ab,kw AND adults:ti,ab,kw
Web of Science	ALL=(nutritional and supplements and oral and health and adults) and Open Access and Articles (Document Types)

### Selection process

The databases were searched by two authors (AR, RS) using the search strategy, which was followed by removal of duplicates, if any. Following which title and abstract screening was performed by two authors (AR, RS) independently, where the authors responded with a ‘Yes’, ‘No’ or ‘Maybe’ for each entry based on the inclusion and exclusion criteria. Any disagreements were resolved with discussion by mutual consensus. The articles included following title and abstract screening, then underwent full-text review by two authors (AR, RS) independently. Disagreements were resolved through discussion with a third author (PD). The articles which qualified at this stage were finalized for quality appraisal and data extraction for this systematic review.

### Quality assessment

Following full-text review, the studies included in this systematic review underwent quality assessment by two authors (AR, RS) independently. Among the four studies included for the present systematic review, three were cross-sectional studies while one was a randomized controlled trial. Therefore, the appropriate version of the Newcastle-Ottawa Quality Assessment Scale ( Wells GA *et al.*, 2021) was utilized for quality assessment of these studies respectively. This risk of bias tool covers three domains i.e., selection criteria, comparability criteria and outcome/exposure criteria, where every study is scored for each domain by giving stars. The selection criteria allotted with maximum of four stars, the comparability criteria with maximum of two stars and the outcome criteria with maximum of four stars.

### Data extraction

The data from the full-text articles was extracted independently by two authors (AR, RS) using a pre-decided format which collected information pertaining to the authors, year of publication, study design, sample size, population studied, intervention or exposure provided, type of nutritional supplement intake, outcome measures, main findings, comparison groups if any, and limitations of the study. The data extracted by the two authors was jointly reviewed and was combined as one to fill out any missing information.

## Results

**Figure 1.**
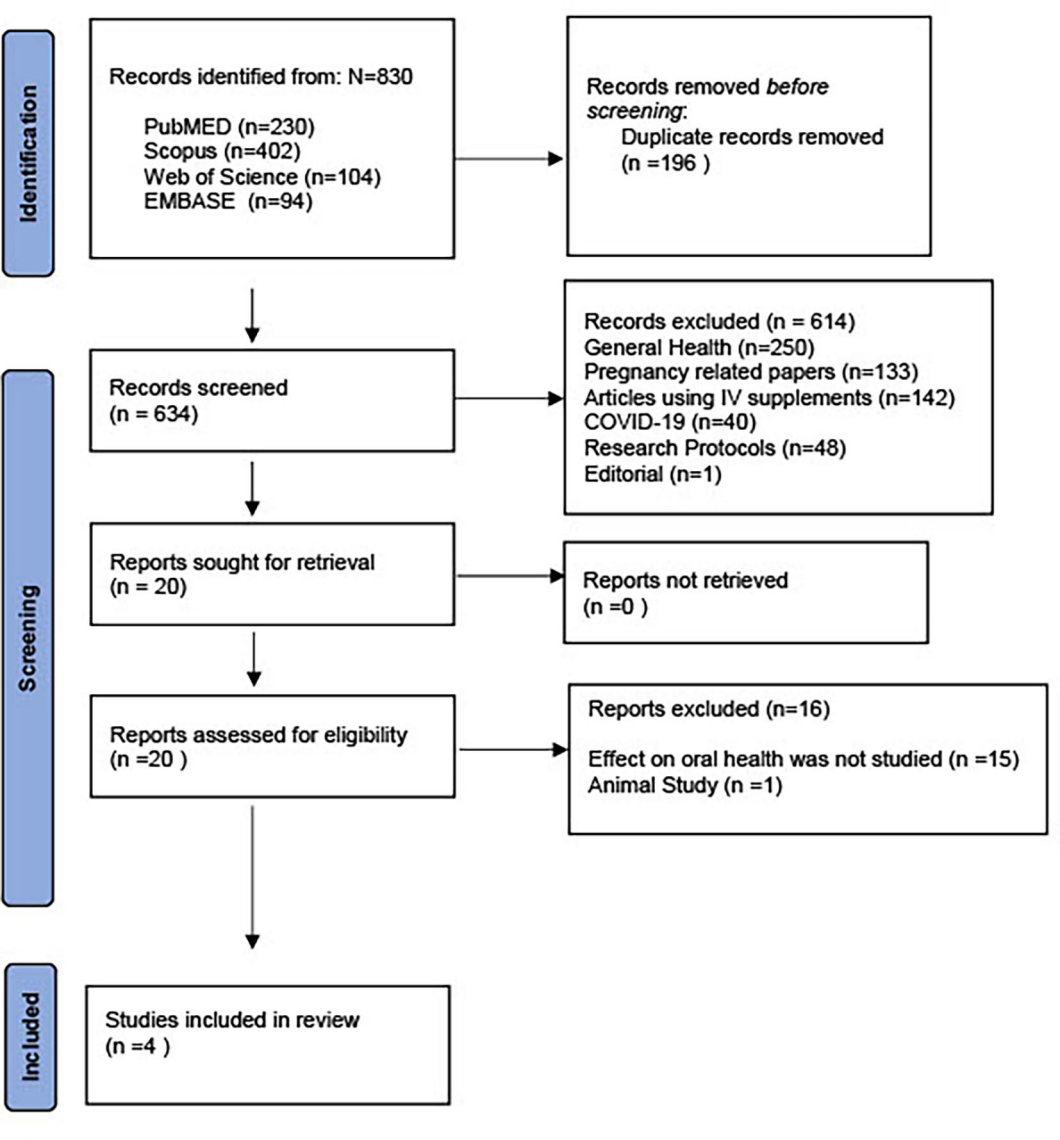
PRISMA 2020 flow diagram for new systematic reviews which included searches of databases and registers only.

The PRISMA flow diagram representing the results of the review process is depicted in Figure 1.

Among the four studies chosen for this systematic review, one was a randomized controlled trial (RCT) while the other three studies were cross-sectional studies. The studies which were selected following full text review underwent quality assessment utilizing the appropriate Newcastle Ottawa Scale and the number of stars allotted to the selected study are depicted in Table 2. The summary of data extraction from these four studies is provided in Table 3.

**Table 2.** Quality assessment of the studies based on Newcastle-Ottawa scale.

Study reference	Selection Criteria ^1^	Comparability Criteria ^2^	Exposure/Outcome Criteria ^3^
Willershausen *et al.*, 2011	***	*	**
Adegboye *et al.*, 2013	*	*	**
Dodington *et al.*, 2015	****	**	**
Luo *et al.*, 2018	****	**	**

Maximum stars means criteria of good quality.

^1^ Maximum of 4 stars.

^2^ Maximum of 2 Stars.

^3^ Maximum of 4 Stars.

* One star.

** Two stars.

*** Three stars.

**** Four stars.

**Table 3.** Data extraction table.

Authors	Study Design and sample size	Population	Intervention or exposure	Type of supplement intake	Outcomes	Main findings	Comparison	Limitations
Willershausen *et al.*, 2011	Randomized Controlled Trial	Dental students(n=42) Mean age – 27.1 ± 3.0 years	Dietary supplements	Orthomol vital f/m; ortho- mol pharmazeutische Vertriebs GmbH.	Dental Assessment Microbiological Analysis Blood Analysis Dietary habits	The group with intake of nutritional supplements had a slight positive effect with lesser increase in C-reactive protein (CRP) levels as compared to the control group. Increase in vitamin C and E and zinc concentration in the blood was observed in the group receiving the nutritional supplement.	_	Small sample size and shorter duration of time.
Adegboye *et al.*, 2013	Cross sectional study	Adult population above 65 years of age (n=606)	Not applicable	Calcium Vitamin D	Oral Examination including number of teeth, unstimulated salivary flow, and plaque score.	There was a significant reduction in plaque score among individuals with higher calcium and dairy servings among those with higher vitamin D intake, after the adjustment for education, gender, age, intakes of sucrose, alcohol and minerals, smoking, number of teeth, diseases, use of dental floss/toothpicks, and visits to the dentists.	_	Higher dropout rates from the original cohort and exclusion due to insufficient data. Lacking detailed data regarding supplemental calcium intake.
Dodington *et al.*, 2015	Cross sectional study	Patients with chronic generalized periodontitis (63 non-smokers, 23 smokers) who were being treated with scaling and root planing participated.	Not applicable	Fruits, Vegetables, β-carotene, Vitamin C, α-tocopherol, Eicosapentaenoic acid (EPA) Docosahexaeboic acid (DHA), α-linolenic acid (ALA), serum 25-hydroxy vitamin D concentrations.	Probing depth and bleeding on probing.	Probing depth was significantly reduced with intake of fruits and vegetables, α-tocopherol, vitamin C, β-carotene, eicosapentaenoic acid (EPA) and docosahexaenoic acid (DHA) intakes in nonsmokers while smokers showed no association.	Smokers and non-smokers.	Small sample size. Self-reported dietary assessment method.
Luo *et al.*, 2018	Cross sectional study	The National Health and Nutrition Examination Survey (NHANES) participants aged more than 30 years with complete periodontal examination	Not applicable	Micronutrients in diet including vitamins A, B1, B2, B6, B12, C, D, E, folate, iron, zinc, calcium, phosphorus, and caffeine.	Periodontal disease and severity.	Severity of periodontal disease increased with decreased intake of Vitamins A, B1, C, E, iron, folate and phosphorus. Second highest level of vitamin D intake was associated with decreased severity of periodontal disease in comparison with highest level of Vitamin D intake.	_	Generalizability of results as NHANES dataset is representative of US population and dietary recall as a method maybe lacking for knowledge of micronutrients.

The nutritional supplements which were studied in the papers included in this systematic review included Orthomol vital f/m; ortho-mol pharmazeutische Vertriebs GmbH (Willershausen *et al.*, 2011) *,* calcium, vitamin D, fruits, vegetables, β-carotene, vitamin C, α-tocopherol, Eicosapentaenoic acid (EPA) Docosahexaeboic acid (DHA), α-linolenic acid (ALA), serum 25-hydroxy vitamin D concentrations as well as micronutrients like vitamins A, E, B1, B2, B6, B12, folate, iron, zinc, phosphorus, and caffeine. ( Adegboye *et al.*, 2013; Dodington *et al.*, 2015; Luo *et al.*, 2018). Only the RCT included in this systematic review included an additional nutritional supplement provided as an intervention, while the cross-sectional studies were based on dietary recall methods such as interviews and questionnaires which also considered additional nutritional supplements if taken by participants along with the dietary nutrients being studied.

The mean age of the participants in the three cross sectional studies was above 50 years, with older adults (65 years and above) being the prime focus in the study by Adegboye *et al.* (2013), while the mean age of study participants was 27.1 ± 3.0 years in the RCT (Willershausen *et al.*, 2011) selected for this systematic review. Among the studies included in this systematic review, the effects of nutritional supplements on oral health were studied via outcome measures such as dental assessment including number of teeth, salivary flow and analysis, plaque score, probing depth, bleeding on probing, microbiological analysis, reduction in periodontal disease and severity.

## Discussion

This systematic review intended to explore the effects of nutritional supplements on oral health in the adult population. The major findings of this systematic review revolved around the positive impact of nutritional supplements on oral structures, plaque scores and general inflammatory processes. A better understanding of the relationship between an individual's diet and oral health can be gained by recognizing the contribution of micronutrients in generating an adequate immune response ( Moynihan, 2005).

Several animal and human studies have very well demonstrated the modulatory role of specific micronutrients on the host's inflammatory response by decreasing inflammatory biomarkers and ultimately bone loss ( Chapple, 2009). A similar trend was observed in one of the studies included in this systematic review by Willershausen *et al.* (2011) where the authors noted that the C-reactive protein (CRP) values, a marker of inflammatory processes, showed a lesser increase among study participants taking the nutritional supplements and a slightly positive effect on gingival inflammation as compared to the control group, where both groups consisted of dental students under high examination stress. In another study included in this systematic review, it was seen that the severity of periodontal disease increased with lesser intake of vitamins A, B1, C, E, iron, folate and phosphorus ( Luo *et al.*, 2018). Although the mechanism of action between nutrition and periodontal disease is not completely understood, Chapple *et al.* observed an inverse relationship between total antioxidant properties and periodontal disease, thereby shedding some light on the complex relationship between nutrition and inflammation leading to periodontal disease ( Chapple *et al.*, 2007). These findings are supported by evidence which recognizes the antioxidant properties of vitamins A, B1, C and E ( Higashi-Okai *et al.*, 2006; Muniz *et al.*, 2015; Palace *et al.*, 1999).

The study by Dodington *et al.* focused on observing the effect of dietary nutrients on periodontal healing among smokers and non-smokers with chronic generalized periodontitis following scaling and root planning. The authors observed a significant reduction in probing depth with higher intake of fruits and vegetables, α-tocopherol, vitamin C, β-carotene, EPA and DHA intakes among non-smokers while no such association was seen in smokers. This association can again be partly due to greater antioxidant intake in the form of fruits and vegetables in the diet. The findings of this study also suggested that whole-food sources, rich in anti-oxidants, are far more beneficial in comparison to supplements with purified compounds in promoting optimal periodontal healing. Beneficial outcomes of EPA and DHA supplements were also observed in participants in this study, which could be explained by the effects of a downstream metabolite of DHA, Resolvin D1, that has exhibited downregulation of inflammatory mediators in an *in vitro* study on periodontal ligament ( Mustafa *et al.*, 2013).

Another included study by Adegboye *et al.* observed protective effects of increased intakes of dietary calcium and dairy products (within recommendations) on the plaque scores among older adults aged 65 years and above with higher vitamin D intakes (more than 6.8 μg/d). This association was presented after adjusting for other confounding factors and participants with lower vitamin D intakes (less than 6.8μg/d) did not demonstrate this association between dietary calcium and plaque scores. This is in line with earlier evidence which is suggestive of an inverse association between higher intakes of calcium, dairy-products and vitamin D with dental caries and periodontitis ( Dixon *et al.*, 2009; Merritt *et al.*, 2006). Since the effect of calcium intake was stratified according to vitamin D in the study by Adegboye *et al.*, it seems that higher intake of vitamin D can improve the positive effects of increased calcium intake, likely by improving the absorption of calcium. This could be explained by the findings of Laky *et al.* who reported that deficiency in vitamin D levels was observed in larger proportions among individuals with severe periodontal disease in comparison with healthy adults ( Laky *et al.*, 2017). In another study included in this systematic review, the second highest level of vitamin D intake (3.2–6.0 μg), was found to be associated with reduced severity of periodontal disease in comparison to the highest level intake (≧ 6.0 μg), which indicates towards an optimal range of 3.2–6.0 μg when considering vitamin D intake ( Luo *et al.*, 2018). This explains the need for further investigation into the optimum levels of vitamin D for oral health promotion and periodontal disease prevention.

The results of the systematic review suggest that developing appropriate dietary interventions focused on oral health, especially periodontal health, might be beneficial alongside routine therapy in prevention of periodontal conditions. These results should be considered with caution due to the limitations of individual studies such as small sample size, high drop-out rates, lack of details regarding supplemental calcium intakes and self-reported dietary assessment method.

### Future direction

The present systematic review retrieved articles from the four databases. The majority of studies included had a cross-sectional design, highlighting the need for more interventional studies in this area. It is necessary to conduct further research to assess the effects of nutritional intervention on individuals with periodontitis, with the goal of developing effective nutritional therapies to manage periodontal inflammation.

## Conclusion

Based on the included literature, this systematic review highlights the beneficial effects of nutritional supplements on oral health when consumed in a recommended dose. These benefits of nutritional supplements were observed in the form of a reduction in plaque scores, gingival inflammation and probing depth and a greater degree of periodontal healing. This systematic review also wishes to highlight the need for interventional studies in this area to explore the effects of nutritional supplements on oral health, especially relating to periodontal healing. The review also stresses the necessity for interventional studies to further investigate the effects of nutritional supplements on oral health, particularly in regards to periodontal healing.

## Data Availability

All data underlying the results are available as part of the article and no additional source data are required. Figshare: PRISMA checklist for ‘Role of nutritional supplements on oral health in adults – A systematic review’. https://doi.org/10.6084/m9.figshare.22709857 ( Shenoy, 2023). Data are available under the terms of the Creative Commons Zero “No rights reserved” data waiver (CC0 1.0 Public domain dedication).
