# Supplementary material for: Role of nutritional supplements on oral health in adults – A systematic review
Source: F1000Res. 2023 May 15;12:492. [Version 1] doi: 10.12688/f1000research.134299.1 (PMC10285321; doi:10.12688/f1000research.134299.1)
Supplement: Peer review history

# Peer review: Reviewer response for version 1

**DOI:** 10.5256/f1000research.147345.r176715

**Published:** 2023-06-20

**Authors:** Suman Challa

**Affiliations:** 1 Department of Comprehensive Dentistry, School of Dentistry, University of Texas Health Sciences Center at San Antonio, San Antonio, TX, USA

The inclusion and exclusion criteria are well-defined. The search strategy is explained, but it would be helpful to know if any restrictions were placed on the language of the publications. They mention quality assessment was performed using the Newcastle-Ottawa Quality Assessment Scale, a well-recognized tool. However, the criteria for the selection of this tool should be explained. The data extraction process is justified, but it would be helpful to have more information on how discrepancies between authors were resolved. The article could benefit from a more detailed presentation of the results, including statistical analyses and effect sizes. The conclusion summarizes the findings but could provide more insights into the implications of the results for clinical practice or policy.

Are the rationale for, and objectives of, the Systematic Review clearly stated?

Yes

Is the statistical analysis and its interpretation appropriate?

Yes

Are sufficient details of the methods and analysis provided to allow replication by others?

Yes

Are the conclusions drawn adequately supported by the results presented in the review?

Yes

Reviewer Expertise:

Oral health in Elders

I confirm that I have read this submission and believe that I have an appropriate level of expertise to confirm that it is of an acceptable scientific standard.

# Peer review: Reviewer response for version 1

**DOI:** 10.5256/f1000research.147345.r176717

**Published:** 2023-06-15

**Authors:** Vijaya Hegde

**Affiliations:** 1 Department of Public Health Dentistry, A. J. Institute of Dental Sciences, Mangalore, Karnataka, India

**General comments:**

 This is an important and well written paper about the usefulness of the Nutritional supplements on Oral health. The Introduction and methodology is well written.

**Specific Comments:**

**Title:** 

- This could be more specific. The mode of delivery of Nutritional supplement could be mentioned and also its impact on which aspect of Oral health.

**Introduction:**

- Definition of ‘Nutritional Supplement’ could be added instead of Diet.
- Briefly mention the current uncertainties of the specific nutritional supplement on Oral health.

**Methods:**

- Kindly address the reasons for excluding the studies.
- Kindly mention if any software was used to extract the data.
- Were any steps taken to resolve inconsistencies?
- How was unclear data/information managed?

**Results:**

- Summary statistics of the studies needs to be mentioned.

**Discussion:**

- Kindly discuss the impact of the study results on policy or further research.
- The Final take away message, needs to be clearly spelled out in the discussion which is missing.
- Where there any limitations in the review process which need to be mentioned?

Are the rationale for, and objectives of, the Systematic Review clearly stated?

Yes

Is the statistical analysis and its interpretation appropriate?

Yes

Are sufficient details of the methods and analysis provided to allow replication by others?

Yes

Are the conclusions drawn adequately supported by the results presented in the review?

Yes

Reviewer Expertise:

Oral Health, Tobacco Induced Diseases, Research and bioethics

I confirm that I have read this submission and believe that I have an appropriate level of expertise to confirm that it is of an acceptable scientific standard.
